# Multiple Sclerosis: From the Application of Oligoclonal Bands to Novel Potential Biomarkers

**DOI:** 10.3390/ijms25105412

**Published:** 2024-05-15

**Authors:** Grazia Maglio, Marina D’Agostino, Francesco Pio Caronte, Luciano Pezone, Amelia Casamassimi, Monica Rienzo, Erika Di Zazzo, Carmela Nappo, Nicola Medici, Anna Maria Molinari, Ciro Abbondanza

**Affiliations:** 1 Unit of Clinical and Molecular Pathology, A.O.U. University of Campania “Luigi Vanvitelli”, 80138 Naples, Italy; grazia.maglio@policliniconapoli.it (G.M.); marina.dagostino@policliniconapoli.it (M.D.); francesco.caronte@policliniconapoli.it (F.P.C.); luciano.pezone@unicampania.it (L.P.); carmela.nappo@unicampania.it (C.N.); nicola.medici@unicampania.it (N.M.); annamaria.molinari@unicampania.it (A.M.M.); 2 Department of Precision Medicine, University of Campania “Luigi Vanvitelli”, 80138 Naples, Italy; 3 Department of Environmental, Biological and Pharmaceutical Sciences and Technologies, University of Campania “Luigi Vanvitelli”, 81100 Caserta, Italy; monica.rienzo@unicampania.it; 4 Department of Medicine and Health Sciences “V. Tiberio”, University of Molise, 86100 Campobasso, Italy; erika.dizazzo@unimol.it

**Keywords:** multiple sclerosis, cerebrospinal fluid, kappa free light chains, novel biomarkers

## Abstract

Multiple sclerosis is a chronic immune-mediated disorder of the central nervous system with a high heterogeneity among patients. In the clinical setting, one of the main challenges is a proper and early diagnosis for the prediction of disease activity. Current diagnosis is based on the integration of clinical, imaging, and laboratory results, with the latter based on the presence of intrathecal IgG oligoclonal bands in the cerebrospinal fluid whose detection via isoelectric focusing followed by immunoblotting represents the gold standard. Intrathecal synthesis can also be evidenced by the measurement of kappa free light chains in the cerebrospinal fluid, which has reached similar diagnostic accuracy compared to that of oligoclonal bands in the identification of patients with multiple sclerosis; moreover, recent studies have also highlighted its value for early disease activity prediction. This strategy has significant advantages as compared to using oligoclonal band detection, even though some issues remain open. Here, we discuss the current methods applied for cerebrospinal fluid analysis to achieve the most accurate diagnosis and for follow-up and prognosis evaluation. In addition, we describe new promising biomarkers, currently under investigation, that could contribute both to a better diagnosis of multiple sclerosis and to its monitoring of the therapeutic treatment response.

## 1. Introduction

Multiple sclerosis (MS) is a chronic autoimmune inflammatory and disabling disorder affecting the central nervous system (CNS) and characterized by demyelination, neurodegeneration, and persistent CNS inflammation [1]. Based on the latest joint project Multiple Sclerosis Atlas, 2.8 million people have MS worldwide. A global increase has been observed in the last decade, essentially ascribed to factors like higher life expectancy, global population growth, enhanced data collection, and improved diagnosis. The prevalence of MS differs among geographical regions and populations, other than by sex and age [1].

Most MS patients have firstly presented a clinical isolated syndrome (CIS), defined as a single demyelinating event affecting the CNS of at least 24 h duration in the absence of other diseases (such as infections, metabolic disorders, etc.) [1,2]. Although MS progression is highly variable among individuals, presenting clinical symptoms with complete or partial recovery followed by periods of clinical stability and/or remission, in most patients, this condition can evolve to Relapsing Remitting MS (RRMS). About a quarter of patients can eventually advance to a progressive MS (PMS) after a long phase of evolution [1,2].

The main cause responsible for the disease is an immune system alteration, particularly involving defects in regulatory T cells (Treg cells). Although MS etiology and the underlying pathogenesis are still debated, recent findings indicate that the contributions of genetic predispositions, infections, and factors leading to pro-inflammatory states, including smoking, obesity, and low sun exposure, provide the molecular basis for its onset [2,3]. For instance, different human leukocyte antigen (HLA) risk alleles as well as variants of the interleukin-2 receptor alpha gene (*IL2RA*) and interleukin-7 receptor alpha gene (*IL7RA*) are likely to be involved [2,3]. In addition, several environmental factors have been hypothesized to participate in the MS etiology. Indeed, many studies have investigated the possible correlation between pathogen infections (certain viral or bacterial antigens or superantigens showing molecular mimicry with self-antigens) or low sun exposure (and, hence, low serum vitamin D) and increased predisposition to MS onset [2,3].

Clinically, a correct MS diagnosis remains a relevant issue considering that many CNS diseases, such as migraine or fibromyalgia, share similar nonspecific symptoms [1], from abnormal magnetic resonance imaging (MRI) to functional neurological disorder and neuromyelitis optica [1]. Currently, McDonald criteria, revised in 2017, represent the most widely employed ones for MS diagnosis [4]. According to these criteria, CNS damage, the presence of plaques in multiple regions of the nervous system, and/or a damage disseminated in time, or occurring at different points in time, should be proven [4]. In addition, the intrathecal IgG oligoclonal band (OCB) presence in CSF can substitute the requirement of demonstrated dissemination in time. Of note, an early therapeutic intervention can delay long-term disease progression and may improve outcomes, which can be classified by the expanded disability status scale (EDSS). Therefore, an accurate diagnosis is needed, especially in CIS patients at high risk of RRMS or PMS developing [1]. Presently, there is no definite cure for MS. Nevertheless, several management strategies are currently available to treat acute attacks, ameliorate symptoms, and reduce biological activity through disease-modifying therapies, thus improving the disability-free life expectancy [5]. Many physicians advise the use of these new therapeutic approaches as first-line treatment for patients with an initial disease stage before permanent disability becomes evident. In addition, it would be also important to evaluate the benefits versus risks of certain therapies [6,7,8]. Indeed, although several therapies are able to reduce the number of relapses, disability increase, and brain MRI activity, especially when a very early treatment is adopted, MS development and the risk of treatment-associated adverse events are extremely variable between individuals. Thus, it is essential to improve a timely diagnosis through the use of new suitable biomarkers to facilitate decisions on the optimal treatment and its onset time to prevent disease progression [5]. 

Since no distinctive clinical features or diagnostic laboratory biomarkers are useful to identify MS with certainty, current MS diagnosis is based on the integration of clinical, imaging (MRI), and laboratory results from CSF analysis [4]. The most used laboratory test evaluates the presence of intrathecal IgG synthesis in the CSF of MS patients. However, several newly proposed CFS and serum biomarkers, useful in the MS diagnosis and prognosis setting at different disease stages, are already experimentally used [2].

Nowadays, CSF analysis for MS diagnosis and the prediction of disease activity after the first demyelinating CNS event is considered of great value. Of note, the OCB detection through isoelectric focusing (IEF) followed by immunoblotting is the current gold standard. This technique analyzes both the CSF and serum samples of each patient. When intrathecal IgG synthesis occurs, OCBs are found in CSF but not in serum [9,10]. This assay has a high sensitivity and specificity of approximately 90% [11]; however, it shows some disadvantages: it only permits a qualitative intrathecal IgG synthesis analysis, and it is technically hardworking, time-consuming, costly, and examiner-dependent [11]. Thus, in the last few decades, several methodological approaches have been developed for intrathecal IgG synthesis detection. Many studies have focused on different quantitative methods measuring IgG concentrations in CSF and serum, then followed these with the calculation of formulae such as that of the IgG index [10]. In addition, both alternative strategies and the search for novel biomarkers, which can help to identify and monitor MS, are continuously under investigation.

This is an overview addressed to all the clinicians in the fields of neurology, biochemistry, and pathology, which are involved in the diagnosis and prognosis of MS. Indeed, we aimed to briefly summarize and update all the available laboratory tools for the MS diagnosis and prognosis, from the current gold standard method to the most promising new biomarker candidates found in the CSF and blood of MS patients, to apply these in the near future. Of note, we also discuss, for the first time, the main forthcoming approaches that are currently under investigation to solve the current issues in this area. We performed the literature search with the PubMed database using, as search keywords, “multiple sclerosis”, “detection methods”, “oligoclonal bands”, “kappa free light chains”, “diagnosis”, “prognosis”, and “novel biomarkers”, alone and/or together, in January 2024. All publications were manually selected for content concerning MS, oligoclonal bands, KFLCs, and novel biomarkers. Of these publications, 93 (60 original articles and 33 literature reviews) were considered for this manuscript.

## 2. Current Biomarkers and Procedures Used in Clinical Setting

### 2.1. The Application of Oligoclonal Bands, IgG Intrathecal Synthesis, and “Gold Standard” Laboratory Test

The relevance of studying the oligoclonal bands goes back to the middle of the last century, when a study reported that 80% of MS patients had elevated gamma globulins in their CSF [12]. From then, this finding was confirmed by further studies [13]. In addition, thanks to the advance of electrophoresis techniques, also, CSF analysis improved quickly; consequently, some specific bands, with the IgG property, could be observed in the γ globulin zone of the MS patient CSF but were absent in the corresponding serum. Hence, a positive correlation between the IgG level in the demyelinating plaques and CSF of MS patients was observed, thus hypothesizing an IgG local synthesis in the brains of MS patients and the designation of these specific IgG bands as OCBs [13,14,15,16]. More recently, the use of IEF has further improved the resolution of protein separation according to the different isoelectric point; this method with specificity on IgG shows high sensitivity and specificity. Therefore, IEF is currently recommended as the “gold standard” to reveal increased IgG intrathecal synthesis [9]. Indeed, this assay allows OCB detection in 95% of MS patients, and OCBs’ presence in the CSF of CIS patients is a prognostic factor of MS conversion, although they could also be found in other chronic inflammatory CNS diseases [13,17]. Commonly, IEF methods are also followed by immunodetection methods (immunofixation or immunoblotting) that have allowed increasing OCB detection sensitivity and specificity [4,13].

**Figure 1:**
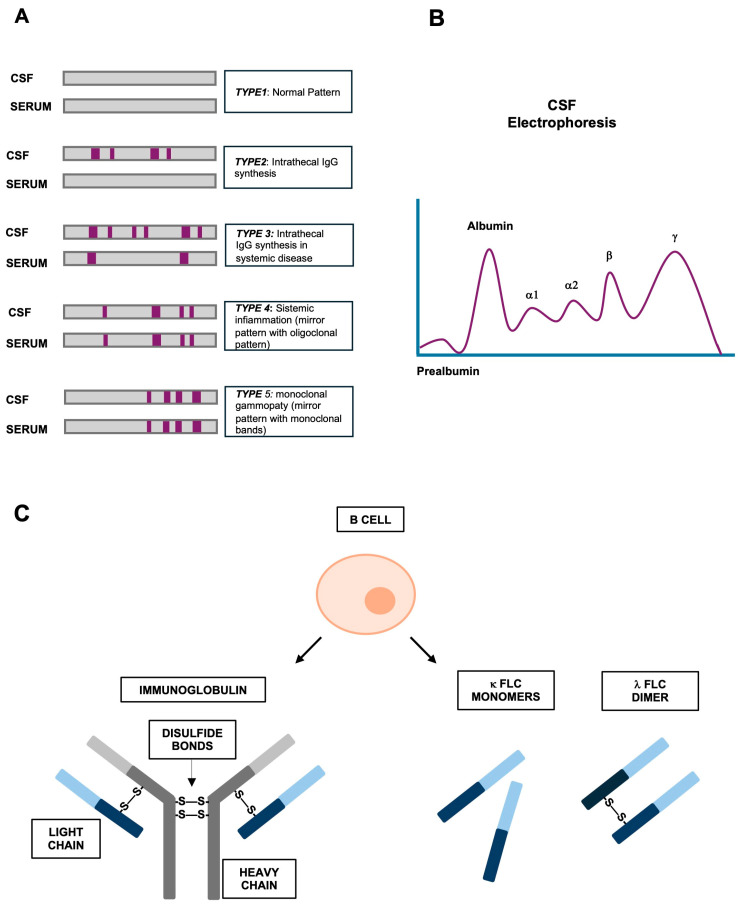
The basis of the currently used MS diagnostic and prognostic methods. (**A**) Different types of IEF patterns. (**B**) Scheme of a CSF electrophoresis of proteins. (**C**) Illustration of immunoglobulin and free light chains (FLCs).

The OCB detection technique requires the parallel IEF separation of paired CSF and serum samples, with a subjective interpretation of results, which can be visualized as five typical OCB patterns for CSF and serum (Figure 1). As mentioned above, intrathecal OCB synthesis is essentially defined as the presence of bands in CSF only or, alternatively, as the presence of bands in both serum and CSF with additional bands in the CSF sample. Moreover, the method standardization is difficult considering that IEF is a multistep and complex procedure with several variables influencing results and high costs [13,18].

Intrathecal positivity of immunoglobulins can be observed also in neuroinflammatory diseases of the CNS other than MS, such as optic neuritis, autoimmune encephalitis, neurosarcoidosis, neuromyelitis optica (NMO), and anti-myelin oligodendrocyte glycoprotein (MOG) antibody disease, and it can be due to several mechanisms including the altered permeability of the blood–brain barrier (BBB), an intrathecal synthesis of immunoglobulins, or a combination of both [13,19,20]. Thus, it is relevant, from a pathophysiological point of view, to distinguish the intrathecal synthesis of immunoglobulins from those entering the CSF from the blood through the BBB. Indeed, during inflammatory processes, B cells can migrate into the CNS and produce immunoglobulins that add to the newly synthesized ones. Therefore, since the results on OCBs only indicate a CNS inflammation, the clinical context should also be evaluated to provide a proper MS diagnosis [13,19,20,21]. Apart from the qualitative OCB determination, IgG production within the CNS can be also evaluated quantitatively through mathematical formulae (IgG index, Reiber formula), which estimate the extra immunoglobulins, adjusted for those caused by the BBB permeability impairment. The simplest and widely used formula is the IgG index formula, which evaluates the CSF IgG amount compared to its serum levels; the index is calculated as the ratio of IgG to albumin in CSF compared to the ratio of IgG to albumin in serum [21,22,23,24]. Albumin is not synthesized in the CNS, and it can be assumed that its presence in the CSF derives exclusively from serum. The CSF albumin concentration is about 200 times lower than that in serum. Analogously, also IgG can migrate from serum to CSF with an average concentration about 500 times lower in CSF than in serum (serum ranges are 37–54 g/L for albumin and 7.0–14.0 g/L for IgG) [22]. Thus, the albumin quotient is included as a dimension of BBB dysfunction in MS [18,24]. Despite its low sensitivity, this method has the advantage of deriving from data (IgG and albumin concentrations) that are routinely evaluated as part of the CSF analysis [18]. Of note, although nearly 70% of MS patients have an increased IgG index, other CNS diseases also display an IgG index increase [21]. The Reiber formulae consider the relationship between CSF–serum concentration quotients of the three immunoglobulins, IgG, IgA, and IgM, and the albumin ratio increase in a nonlinear manner with progressive BBB impairment [19,21]. In the presence of such damage, this formula yields fewer false-positive results than the IgG index. Similarly, a more recently proposed approximation formula (Auer’s formula) is likely to produce few false-positive results, especially for IgM and IgA, whose detection has, however, very limited purposes in routine CSF analysis [25]. Interestingly, a recent retrospective analysis applied the linear IgG index [22], the Auer’s formula [25], and the hyperbolic Reiber’s function [21] on a cohort of 372 patients with CNS demyelination to evaluate their performance characteristics in determining the intrathecal IgG synthesis [26]. This study revealed that despite a high specificity showed through the Auer’s method (95%), this formula had a very low sensitivity compared to Reiber’s one and the IgG index (68% versus 83% and 89%, respectively), suggesting an overall superiority of the hyperbolic function.

Similarly, IgM OCBs can also be detected as result of intrathecal IgM production, which has been linked to an increased risk of disease progression [27]. Likewise, the IgM index, which can be calculated using the same formula as the IgG index, predicts a poor disease prognosis [28].

Overall, despite its recognized validity, OCB detection through IEF combined with immunodetection is not a disease-specific procedure and should be evaluated together with other disease clinical phenotypes; moreover, a negative result does not exclude MS. Technically, OCB is a complex method that exhibits several methodological problems. Firstly, it requires proper interpretation by specialized operators, thus lacking objectivity and standardization; moreover, it provides only a qualitative but not a quantitative result. In this context, additional objective, and complementary diagnostic tools, such as intrathecal synthesis rates of immunoglobulins, could support MS diagnosis, help in differentiating between MS and other neuroinflammatory diseases, and be useful for follow-up and prognosis. Thus, many efforts have been produced to establish the optimal mathematical formula to measure intrathecal immunoglobulins, with Reiber’s hyperbolic function having been recently recommended for its superior accuracy performances.

Based on these findings, further CSF biomarkers have been investigated and proposed so far, as discussed below.

### 2.2. Kappa (and Lambda) Free Light Chains as New Biomarkers for MS Diagnosis

During antibody synthesis, B lymphocytes produce intact immunoglobulins with light and heavy chains bound together via disulfide bonds and noncovalent interactions, and also free light chains (FLCs) in excess over heavy chains, and secrete them into the blood circulation [29,30] (Figure 1C). These FLCs consist of two immunoglobulin domains, a constant region that specifies the isotypes of free light chains (either κ or λ), and a variable domain; κFLCs exist mainly as monomers whereas λFLCs are present as covalent dimers [29]. Although their function has not yet been fully elucidated, it is well recognized that an increase in both FLCs (κ and λ) can be found in patients with inflammatory and autoimmune systemic diseases including MS [29,30]. Thus, in the last few years, many studies have proposed their possible use as biomarkers for intrathecal B cell activity in MS patients, mainly to replace immunoglobulins, especially for those patients with signs and symptoms suggestive of a demyelinating event but with no clear IgG band [31].

FLCs, originally identified in 1847 and called Bence Jones proteins, represent a relevant biomarker in clinical laboratory diagnostics [32]. It is noteworthy that in 1980, the kappa free light chain (KFLC) concentration was measured in the serum and CSF of MS patients using a nephelometric assay [33]. This method has been successively automated, and it remains one of the most used to measure KFLC, together with turbidimetry and ELISA (enzyme-linked immunosorbent assay) (reviewed in [34]). Few studies have also compared these different techniques or diverse instruments while applying the same method but with essentially similar results in terms of diagnostic sensitivity and specificity [34,35,36]. However, an ELISA assay based on monoclonal anti-kappa and anti-lambda antibodies against cryptic FLC epitopes revealed better results than the nephelometric assay [37]. In addition, other determination methods, such as affinity-mediated immunoblotting after isoelectric focusing, have also been tested and proposed as useful alternative strategies [35,38]. It is noteworthy that although different automated nephelometric assays are currently available for FLC detection with comparable performance, the same assay should be preferably employed during patient follow-up [39]. The cut-off for the CSF KFLC concentration varied between 0.103 μg/mL and 7 mg/L and a mean diagnostic sensitivity of 86% with 95% confidence interval (80%, 92%) and specificity of 91% and (86%, 96%) as confidence interval [34].

Of note, an important issue that limited the past use of FLCs was the difficulty in yielding FLC-specific antibodies without cross-reactivity with light chains of complete immunoglobulins. Thus, a great step forward was also represented by the development of FLC-specific detection antibodies against epitopes that are hidden in intact immunoglobulins [40].

It is generally recognized that the values of both κFLCs and λFLCs increase in the CSF of MS patients compared with patients of non-inflammatory neurologic diseases; however, the relative λFLC increase was found to be less than that of κFLCs [37]. In addition, a previous study reported that MS patients had higher κFLC levels but similar λFLC levels as compared to patients with CNS infectious diseases, thus suggesting that elevated λFLC could be considered a potential infection marker rather than MS one [41]. Even more recently, no reliable data could be obtained for the λ-index since λFLCs were often below the detection limit and therefore could not constitute a valid test for CIS/MS diagnosis [42,43]. Overall, these findings indicate that κFLCs show better correlation in MS diagnosis and their index has higher sensitivity and specificity than λFLCs.

Altogether, some studies utilized κFLC concentration levels to assay intrathecal synthesis and described high diagnostic accuracy although with a certain variability of the range values because of both the diverse technologies used as well as the different patient populations included, in terms of both analyzed case number (mostly small populations) and heterogeneous groups (for instance, control groups comprising inflammatory neurological disease patients). In addition, as for immunoglobulins, κFLCs can be measured using further calculation methods (as discussed below).

### 2.3. κFLCs in the CSF of MS Patients and Early Disease Activity

The first studies determining κFLCs in MS diagnosis did not take into consideration the integrity of the BBB and inter-individual concentration variations, thus generating false-positive and -negative results. Instead, as for IgG, it is essential to determine the amount of locally synthesized κFLC fraction in the CSF. Thus, in the last years, the most common approach has been the implementation of intrathecal κFLC determination, and most laboratories have begun to evaluate the κFLC index, which is the ratio between CSF and serum κFLC levels, considering the altered BBB permeability through the CSF/serum albumin quotient (Qalb), and can be calculated using a formula (κFLC_CSF/κFLC_Serum/Qalb) (Table 1) [43,44].

To date, results from many studies have established that the κFLC index reaches a high diagnostic accuracy to identify MS patients. As reported in a very recent meta-analysis, the diagnostic sensitivity of the κFLC index ranges from 52 to 100% (weighted average, 88%) and specificity ranges from 69 to 100% (89%) whereas for OCBs, sensitivity ranges from 37 to 100% (85%) and specificity from 74 to 100% (92%) [45]. The mean differences in diagnostic sensitivity and specificity between the κFLC index and OCBs were +2 and −4 percentage points; no difference was observed for the diagnostic accuracy [45]. Strikingly, the observed wide range of values for both the κFLC index and OCBs derive from the heterogeneity in the patient groups between studies, especially if patients with inflammatory neurological diseases were included into the control group.

Nevertheless, a better approach to measure intrathecal κFLC synthesis still needs to be established [34,46,47]. Indeed, to date, most studies have used the absolute CSF κFLC concentration, κFLC quotient (Q-κFLC: CSF κFLC/serum κFLC), or κFLC index (as defined above) [34,48]. In addition, further algorithms have been proposed and are currently under investigation (Table 1) [34,35]. These methods calculate the intrathecal κFLC fraction (IF_κFLC) through different formulae determining the QAlb-dependent predefined upper reference limit [49,50,51,52]. Among them, the most recommended is the Reiber diagram since it is based on pathophysiology and has low influenceability from pre-analytic factors and because of its outstanding accuracy performance [10,34,45].

The rationale about the use of absolute CSF κFLC concentrations was that the contribution of FLCs derived from blood to the total concentration is very low in pathological conditions with intrathecal synthesis; indeed, the intrathecal κFLC fraction is greater than 80% in most MS patients [53,54]. However, the impact of serum κFLC levels and the Qalb could be significant in patients with only low or modest intrathecal κFLC production. Moreover, it is now clear that some confounders, like elevated serum FLC levels or a high degree of BBB dysfunction, may also occur [48]. Indeed, a study reported different κFLC index values between MS converters and nonconverters whereas the absolute CSF κFLC concentrations were similar between the groups, thus highlighting the importance of determining intrathecal κFLC synthesis by calculating the κFLC index [55]. Of note, all these mentioned parameters have been reported to show a diagnostic accuracy for MS diagnosis similar or superior to the presence of OCBs, even in the prediction of inflammatory or infectious CNS diseases [34]. In a recent meta-analysis, several different κFLC measures were considered and compared with OCB detection results even though statistically sufficient power was found only for the κFLC index so that the potential use of the other measures could not be demonstrated [45]. Anyway, all the approaches were able to determine the presence of the intrathecal synthesis of kFLCs when they were elevated while the kFLC index maintained high diagnostic performance even in the case of low and borderline values [10,45].

Accumulating evidence suggests that FLCs also have prognostic value. Indeed, the intrathecal κFLC synthesis is associated with conversion from CIS to clinically definite MS, and the κFLC index may predict the conversion time to MS and disability progression [37,46,56]. However, it is still unclear whether this prognostic value remains valid when adjusting for other prognostic factors through a multivariate approach. This issue was recently challenged in a study on 88 patients with a first CNS demyelinating event that were followed for a period of 4 years [55]. In this study, a multivariate analysis performed after the first demyelinating event revealed an increase in the κFLC index by 10, indicating an increased risk for a second clinical attack, and its probability within 12 months in patients with an elevated κFLC index was higher than in patients with a low κFLC index [55]. These results are of great value for clinical practice since they could provide a reliable biomarker to identify patients with early MS and a higher risk for further disease activity, who could benefit from the use of highly effective therapies.

**Table 1:** Parameters/algorithms for KFLC measurement.

Parameter/Algorithm Used for κFLC Measurement	Formula
CSF κFLC concentration	Absolute concentration
κFLC quotient (Q-κFLC)	CSF κFLC/serum κFLC
κFLC index	Q κFLC/QAlb
* Presslauer’s formula [49]	QLim-FLC = 0.9358 × QAlb^0.6687^
* Hegen formula [50]	QLim-FLC = 3.1276 × QAlb^0.8001^
* Reiber’s formula [51]	QLim-FLC = 3.27 (QAlb^2^ + 33)^0.5^ − 8.2 × 10^−3^
* Senel’s formula [52]	QLim-FLC = 14.85 + 2.41 × QAlb

QAlb: CSF/serum albumin quotient. * These methods calculate the intrathecal fraction through different formulae determining the QAlb-dependent upper reference limit.

### 2.4. κFLC Index: Advantages and Open Issues

Overall, the literature data suggest that κFLC index determination has several important advantages when compared to OCB detection, which currently represents the gold standard technique. Although no relevant differences in diagnostic accuracy have been found, the method to measure the κFLC index is easier, more reliable, and less expensive and time-consuming. Indeed, an estimated OCB determination requires about 3–4 h while nephelometric and/or turbidimetric assays take 20–40 min. In addition, serum and CSF IgG concentration analyses are necessary to carry out the correct dilutions for the OCB evaluation. On the other side, following the dosage of both serum and CSF IgG, albumin, and κFLC, the related indexes can be calculated without additional costs. Furthermore, the personnel who carry out the OCB evaluation must be highly specialized, and since it is a semi-automatic technique, the operator is forced to follow all the phases whereas nephelometry and turbidimetry are completely automated and non-operator-dependent methods. Of note, from a technical point of view, the determination of κFLC in the CSF and serum requires a sample volume of at least 200 μL (due to the dead volume in the cuvette placed in the nephelometer or turbidimeter). On the other side, OCB testing requires only a minimum of approximately 10–20 μL (loaded on the gel for IEF); however, as mentioned above, the determination of IgG concentration is recommended before the IEF run [10].

Moreover, in contrast to OCB detection, the determination of the κFLC index is objective, being operator-independent, and quantifiable since it provides a metric result covering a range from approximately 1 up to 500, which is useful for predicting disease activity [53,55,57]. 

Although the κFLC index may represent a potential diagnostic biomarker due to the promising results, the high sensitivity and specificity, and the important technical advantages compared to OCB detection, some topics need to be refined before its use in the clinical routine setting. Mainly, the κFLC index cut-off should be defined since there is no consensus about its diagnostic values because of their high variability among studies.

Notably, the κFLC index cannot predict whether increased κFLC measures are the consequence of an intrathecal IgA, IgM, and/or IgG synthesis. CSF κFLC levels do not provide information on the clonality of immunoglobulin production and do not allow a differentiation between systemic inflammation with an additional intrathecal inflammation and an isolated intrathecal inflammation. This further information can be provided by OCBs and can be helpful in some clinical situations, such as in patients with monoclonal gammopathies and suspected CNS involvement or with the CNS involvement of systemic diseases. Among the main limitations of κFLC index determination, there is also the existence of different formulae for calculating it. Thus, cut-off values might apply depending on the clinical question, for example, to provide an upper reference limit determined in a control (non-inflammatory) population or to differentiate MS from other inflammatory neurological diseases. Furthermore, cut-off values might vary based on whether the main aim is to increase diagnostic sensitivity or specificity [45]. The definition of cut-offs should facilitate the diffusion of the κFLC index, but it is obviously, as already reported, difficult to determine whether the index could be used to improve diagnosis, prognosis, and therapeutic decisions and in which way [48].

Of note, as discussed above, the best option to measure intrathecal κFLC synthesis still needs to be established [34,46,47]. In this context, however, the laboratory method and the approach applied for κFLC measurements might also influence the choice of cut-off points. In addition, the difficulty in establishing the optimal cut-off points for MS diagnosis essentially depends on the heterogeneous control populations that have been examined so far. Indeed, standard values might be different based on whether non-inflammatory control individuals or patients with other inflammatory neurological diseases are considered as controls; thus, different cut-off values might be useful [58]. Currently, κFLC index values are quite different among these diseases, displaying the highest values in MS followed by other inflammatory neurological diseases and then by non-inflammatory diseases or healthy individuals.

For instance, in a recent study measuring several κFLC parameters, a 7.25 cut-off for the κFLC index showed better sensitivity (85% vs. 77%) but less specificity (88% vs. 91%) than OCBs [57]. This diagnostic performance was in accordance with many previous reports showing higher sensitivity but lower specificity than OCBs [43,46,59,60,61,62,63,64,65]. Overall, these findings indicated that the lack of high κFLC index values is more powerful for excluding MS than negative OCBs. In this study, one MS patient with positive OCBs showed a κFLC index below the diagnostic cut-off of 7.25. Therefore, a threshold of ≥2.55 (97% sensitivity) was suggested to reduce OCB analyses by 52% of the studied samples. Likewise, rarely, MS patients showed very low κFLC index values and negative OCBs, particularly CIS cases, as observed in this study (one case < 2.55) [57]. Otherwise, a previous study had suggested a lower threshold (0.92) for the performance of OCB testing with a similar sensitivity (97%) [66]. It is noteworthy that a further recent study established an algorithm based on the use of three different κFLC index cut-offs to help the probability evaluation of MS at diagnosis and before the beginning of therapy [67]. Specifically, a κFLC index < 3.3 is strongly associated with a low MS risk, as has already been reported [61], and OCB evaluation is not required under this cut-off for a differential diagnosis (negative predictive value for MS > 99%). Instead, very high values (>55) are strongly associated with MS and/or may be indicative of shortened disease-time progression for CIS patients (specificity 100%); even in this case, OCB analysis can be avoided. An optimized cut-off of 9.1 with better sensitivity and equivalent specificity than OCB for the MS diagnosis can distinguish moderate (range 3.3–9.1) from high (range 9.1–55) probability of MS, not higher than the probability of having other inflammatory neurological disorders; in both cases, OCB testing is necessary [67]. This agrees with previous studies that used κFLC index cut-off values from 8 to 10 [46,68]. It is noteworthy that a recent meta-analysis reported cut-off values for the κ-FLC index ranging from 2.4 to 20 with a discriminatory cut-off at 6.1 to differentiate CIS/MS patients from controls [45].

Interestingly, few studies have investigated the use of a prognostic cut-off to select patients with a major relapse risk, potentially influencing initial therapeutic decisions. For instance, a study proposed a κFLC index cut-off ≥ 106 as a prognostic cut-off to identify subjects at risk of the worst MS course [68]. Similarly, a previous report selected patients at the first clinical episode and divided them into two subgroups defined as “high” or “low” based on the established κFLC index cut-off value of 102.5; the average index in the two subgroups was used in the Cox regression, demonstrating that subjects with a κFLC index > 100 had a higher probability of progression to clinical definite MS [55].

Finally, studies that address κFLC index reproducibility using different assays, platforms, and cut-offs between centers are also required, even though some findings are for absolute serum κFLC concentrations.

Overall, the κFLC index has great potential to be a promising biomarker that could be used as a screening test before OCB detection and eventually replace its analysis. Indeed, it shows a high accuracy, like OCB analysis, but it also has significant methodological advantages. In addition, its putative prognostic value could be useful to identify early MS patients with a higher risk for further disease activity for which early and highly effective therapies could be recommended to prevent long-term disability and/or to significantly delay disease progression. Thus, further studies are essential to validate the independent prognostic value of the κFLC index in early MS.

## 3. Future Perspectives: The Search for Novel Potential Biomarkers

**Figure 2:**
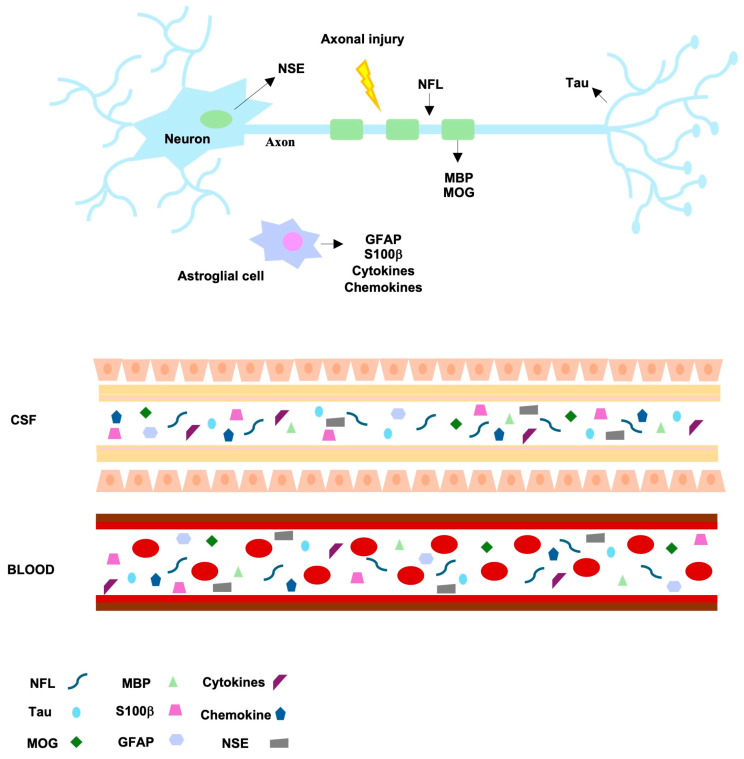
New MS biomarkers under study for clinical validation: schematic illustration of their involved mechanisms and biological fluids (CSF and blood). Shown biomarkers: NFLs (neurofilament light chains), Tau protein, MOG (oligodendrocyte glycoprotein), MBP (myelin basic protein), S100β protein, GFAP (glial fibrillary acidic protein), NSE (neuron specific enolase), cytokines, and chemokines.

As discussed, MS presents a highly variable course among individuals, ranging from reversible symptoms to permanent disability [1,2], and the early use of disease-modifying therapies can significantly influence its progression although with potential side effects [5]. Thus, the identification of early diagnostic and prognostic biomarkers is critical for providing tailored and adequate treatments. Currently, several MS candidate biomarkers that could be used in the near future to predict, diagnose, correlate with disease, and monitor the response to therapeutic treatments are under investigation. Overall, they are involved in various pathological processes such as demyelination, glial dysfunction, axonal and neuronal damage, and pro-inflammation (Figure 2) (reviewed in: [2,69,70,71]).

The most studied emerging MS biomarker is represented by neurofilament light chains (NFLs), which can be assessed quantitatively and can be easily detectable in both CSF and serum/plasma. NFLs are scaffolding proteins of the axonal cytoskeleton that, during axonal damage, are released in high quantity into the extracellular space, CSF, and serum; hence, their concentration levels indicate the axonal damage extent, therefore constituting a biomarker of neurodegeneration [72]. Although cerebrospinal NFL has shown low specificity for MS diagnosis [72], a consistent correlation has been observed between serum NFL and disease activity and therapy and a variable association with disability [73]. However, the possible influence of comorbidities and age has limited the diagnostic use of this biomarker in clinical settings, highlighting the need for the definition of appropriate ranges of threshold values [2,70,74].

Further promising biomarkers implying demyelination, glial dysfunction, and axonal and neuronal damage include, among others, Tau protein, glial fibrillary acidic protein (GFAP), S100β protein, myelin basic protein (MBP) and myelin oligodendrocyte glycoprotein (MOG), and neuron specific enolase (NSE) [2,69,70,71]. Overall, the use of these markers is limited by similar issues as those of NFLs; indeed, they indicate a damage that can also be uncovered in other neurodegenerative disorders; moreover, conflicting results between different studies have emerged [70]. Hence, a prospect could be represented by the combined use of several biomarkers. Importantly, to date, the laboratory tests commercially available for MS diagnosis include, together with OCB and kFLC measurements, the quantification of NFL and GFAP levels, which are accessible through patient serum. However, despite being commercially available, these laboratory tests have not gained widespread approval in clinical practice, being mainly of prognostic value rather than serving as diagnostic tools [75].

Since MS is a chronic immune-mediated disease characterized by chronic inflammation, the activation of B and T cells produces the release of numerous pro-inflammatory cytokines, which could be useful in MS diagnosis and in the evaluation of disease progression. For instance, in a study, serum IL-6 levels were detected at a higher rate in MS patients vs. controls and were correlated with the age of onset for MS patients [76]. Furthermore, MS patients overexpress IL-15 in both serum and CSF [77]. Likewise, another studied biomarker is the chemokine CXCL13, which is significant in the conversion from CIS to MS as it activates B and T helper cells in a process involving its receptor CXCR5 on demyelinated lesions [78]. Additional promising immune mediators comprise plasma soluble CD40L (sCD40L), chitinase-3-like-1 precursor (CHI3L1), the heat shock proteins (HSPs) HSP70 and HSP90, and osteopontin (OPN) [2,69,70,71]. It is noteworthy that several studies are ongoing to establish whether certain immune signatures could distinguish MS patients in the early stage of the disease from patients suffering from other inflammatory and non-inflammatory neurological disorders [79] or to further differentiate underlying disease pathogenesis and disease activity.

An important research field also focuses on the measurement of the currently available biomarkers (like FLCs) in alternative biological fluids other than CSF and serum, including urine, saliva, and tear fluid, which can be easily obtained without invasive procedures and, therefore, facilitate the biochemical follow-up of patients and/or therapeutic monitoring. Despite some encouraging data, their diagnostic utility in the clinical setting of MS patients still needs to be validated [79,80,81,82].

**Figure 3:**
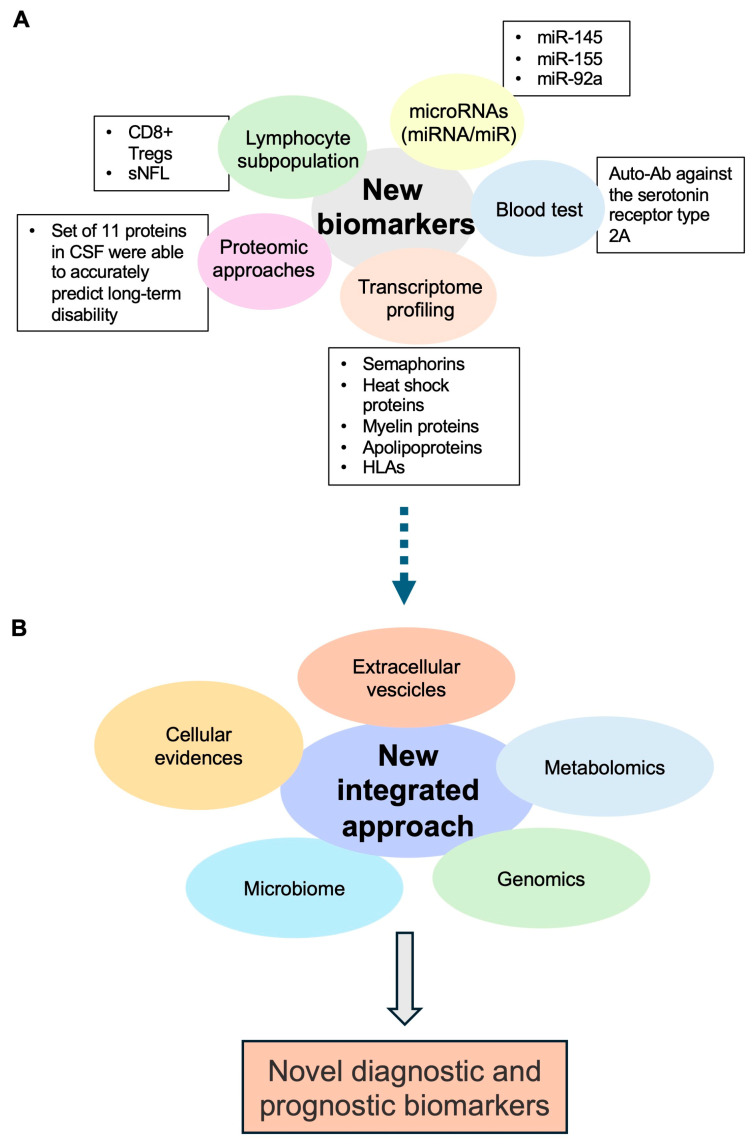
New potential MS biomarkers. (**A**) Novel identified biomarkers currently under investigation for their validation and use in the clinical practice, including the study of lymphocyte subpopulations, autoantibodies, several microRNAs (miRNA/miR), and differentially expressed genes or proteins. (**B**) A new integrated approach involving several methods (i.e., cellular evidence, extracellular vesicles, metabolomic approaches, and microbiome and genomics methods) to identify additional diagnostic and prognostic MS biomarkers.

In the last few decades, transcriptome profiling has been used to investigate human diseases at a molecular level and to search for novel molecular biomarkers and therapeutic targets in many human diseases including MS [83]. As for other diseases, the number of newly discovered molecular markers and key drivers of MS disease progression is continuously increasing thanks to the whole-expression studies obtained through bulk- or single-cell analysis using RNA sequencing strategies [84]. In addition, a future integrated bioinformatics approach combining cellular, metabolomics, microbiome, genomics, proteomics, and extracellular vesicle studies will allow the identification of new diagnostic and prognostic biomarkers (Figure 3).

Although very few “omics” studies have been performed on MS brain tissues, they have revealed that MS is a brain disease wherein all cells are altered at different degrees. In addition, the findings of these analyses have shown that MS is a very complex and heterogeneous disease at a molecular level when compared to the currently used clinical classification [85]. Overall, gene expression increases in semaphorins, heat shock proteins, myelin proteins, apolipoproteins, and HLAs have been observed. Furthermore, different lesions have been characterized by distinct astrocytic and microglial polarization, altered oligodendrogenesis, and changes in specific neuronal subtypes. White matter lesions have expressed high levels of *CXCL12*, *SCD*, and *CD163* and an activation of STAT6- and TGFβ-signaling pathways. Otherwise, grey matter lesions have shown TNF signaling alterations driving cell death and neurodegeneration, especially in *CUX2*-expressing neurons [80].

Currently, several transcriptome data have also been obtained from the CSF, blood, or peripheral blood mononuclear cells (PBMCs) of MS patients. As expected, most of the relevant studies were conducted on blood or on PBMCs that are quite easy to obtain; additionally, it is well known that T, B, and NK cells are involved in MS triggering or progression. Thus, the knowledge of whole-transcriptomic changes in MS patient blood cells may lead to the identification of possible altered networks even after specific treatments targeting the immune response (reviewed in [86]). Of note, the analysis of PBMCs has allowed studying the transcriptome profiles of different lymphocyte subtypes, especially through single-cell RNA-seq [87]. Moreover, in a study, different gene expression profiles were found during treatments with interferon (IFN)-β1 in men and women. Likewise, blood analysis showed interesting results in MS at various disease stages. For instance, the gene expression in MS relapse was significantly changed at night compared with either relapse during the day or MS in remission [86]. Taken together, these findings highlight the importance of transcriptomic analysis for personalized medicine since it could provide a useful tool to stratify MS patients based on their gene expression signatures before therapeutic treatment.

Moreover, many studies have shown that several microRNAs (miRNA/miR), which play a fundamental role in gene regulation by targeting mRNAs, are able to control the expression of genes involved in MS pathogenesis. These studies have revealed some differences in miRNA profiles in blood or other biological fluids of MS patients and healthy controls, as well as among MS subtypes or with diverse outcomes or therapeutic responses [70,88,89]. For instance, despite the conflicting results, some miRNAs involved in inflammatory signaling and the regulation of lymphocyte subsets have been identified, such as miR-145, miR-155, and miR-92a, thus suggesting their possible future use as reliable indicators of MS activity [70,88,89].

However, these results need to be confirmed on large cohorts of MS patients, and they should be integrated with other omics data. Indeed, proteomic approaches have also become important tools to understand MS pathogenesis and yield candidate biomarkers for clinical utility in the near future [90]. Interestingly, a recent study used a highly sensitive and specific technology combining proximity extension assay with next-generation sequencing (PEA-NGS) to measure the expression of 1463 proteins in paired CSF and plasma samples from two cohorts of individuals with early-stage MS and healthy controls. This strategy led to the identification of a set of differentially expressed MS-relevant proteins, which could be used to predict, either individually or in combination, short-term disease activity and long-term disease progression [91]. Particularly, a set of 11 proteins in CSF (CXCL13, LTA, FCN2, ICAM3, LY9, SLAMF7, TYMP, CHI3L1, FYB1, TNFRSF1B, and NFLs) were able to accurately predict long-term disability; additionally, NFLs were confirmed as robust markers of short-term disease activity. Of note, some of these proteins, such as CXCL13, LTA, SLAMF7, CHI3L1, and NFLs, are already recognized MS biomarkers for prognosis and treatment response assessment [91].

Another emerging field is the study of the altered lymphocyte subpopulation. Indeed, a very recent study has demonstrated, for the first time, an association between MS disease severity and the functionality of CD8+ Tregs [92]. Additionally, a moderate correlation between sNFLs and lymphocyte cell subpopulations has been shown in an exploratory analysis, thus confirming that sNFLs constitute a biomarker of disease worsening and suggesting that circulating immune-cell subset frequencies combined with sNFL concentration could be used as potential prognostic markers in MS if confirmed through additional studies [93].

Antibodies towards neuronal surface antigens could be involved in numerous neurodegenerative diseases including MS. Indeed, a potential link has been suggested between some autoantibodies (Aabs), such as Anti-MBP and Anti-MOG Aabs, and MS onset and/or progression [94].

It is noteworthy that a very recent study has reported the development of an ELISA test to reveal the presence of an MS-specific auto-Ab against the serotonin receptor type 2A in blood samples, with a sensitivity and specificity above 95%. This assay was set up due to the contribution of the serotonin pathway in MS and the use of the 5-HT receptor subtype 2A as a virus gateway to induce a demyelinating disease [75].

## 4. Conclusions

To date, a single laboratory test showing a sufficient performance to diagnose MS is not available; both OCB detection and the κFLC index are very sensitive tests in MS prediction. Since the latest McDonald’s criteria revision for MS diagnosis [4], the gold standard has been OCB determination in the CSF. However, very recently, κFLC index determination has been acquiring ever more relevance, representing a simple, reliable, fast method that allows saving time and costs. In addition, the κFLC index shows a high specificity like OCB detection but with variable sensitivity due to different cut-off values. Currently, one of the main limitations of this biomarker for its introduction into clinical practice is the lack of a standardized cut-off value that allows to differentiate both MS and healthy patients and patients affected by other neurodegenerative diseases. Thus, the κFLC index, characterized by rapid performance, low costs, and clinical validity, could be used as the first screening test and the use of OCB detection as a confirmatory test. The integration between patient signs and symptoms, instrumental examinations, and the use of laboratory combined assays could be useful to obtain an early and certain MS diagnosis. Nevertheless, further studies are needed to identify and/or validate novel molecular biomarkers to improve the diagnosis, follow-up, and monitoring of MS.
